# Introductory

**Published:** 1883-01

**Authors:** W. C. Barrett

**Affiliations:** Buffalo, N. Y.


INTRODUCTORY.—It is with unfeigned reluctance, and only after a considerable period of hesitation, that the undersigned assumes editorial charge of the Dental Department of the INDEPENDENT PRACTITIONER. A busy man, already sunk to his neck by the weight of personal responsibilities and professional cares, it seems mere presumption in him to assume to lead when he should only follow. Yet ever since his connection with a profession to which he has devoted his life he has been a contributor to its literature, and it is perhaps as well that he should finally try the experiment of standing alone, instead of always remaining in editorial leading-strings. He therefore undertakes the labor, determined to devote to it all the energy of which he is capable. A place in the literature of an honorable profession is an enviable position, but he is well aware that it is not to be won at a single stride.

It is but reasonable that the readers of the INDEPENDENT PRACTITIONER should be taken into editorial confidence and given an inkling of what our hopes and aspirations are. The journal has now been established sufficiently long to give promise of a continued existence, and it should be understood that it has a definite aim. Unconnected as it is with the commercial element of the profession, it must stand upon its merits alone. It has no dental depot, no great manufacturing interests, no advertising demands to sustain it. It must rely upon its entire freedom from all such alliances, and as an independent journal appeal to the profession for support.

The Dental Department of the INDEPENDENT PRACTITIONER will be kept abreast the advance of thought. At the present time the tendency in investigation is largely toward the etiology of dental diseases, and in this field we hope it will do some effective work. A spirit of questioning is abroad in the land, and dentists are inquiring into the verity of traditions in which they have been long disciplined. Practitioners are audaciously catechising their quondam preceptors, and making deep study to find upon what foundation old-established theories rest. It is a disputatious and a sceptical generation. Still, endeavoring to discriminate between zeal and zealot, between honest investigation and wanton iconoclasm, the INDEPENDENT PRACTITIONER will do all in its power to aid original investigation, and it invites free expression of opinion from its correspondents upon all that is new in dentistry.

There is a large class of diseases whose pathology and treatment lie upon the border land between general medicine and dentistry, but the results of which may extend into and involve tissues and organs that are beyond any debatable ground in the undisputed practice of either. It is of that class of disorders that this journal will be specially mindful. Appealing as it does to both a medical and a dental constituency, it should be especially the organ of such as practice dental *medicine*, and its Dental Department will therefore pay particular attention to the materia medica and the therapeutics of the profession. These are subjects which have been too much neglected, and as a consequence our pharmacopœia is far too limited. Too large a proportion of dentists are quite content with half a dozen, or even less, of topical remedies. They do not subscribe for any medical journal, and their opportunities for extending their knowledge in this department are insufficient. To those dentists, therefore, who desire to keep fully posted in the medical department of their profession, and to those medical men who have an interest in the dental department of medicine, the INDEPENDENT PRACTITIONER will be a welcome visitor.

Finally, as the undersigned has, so far as ability went, ever been a liberal contributor to the literature of his profession, he with the greatest confidence appeals to his brother practitioners for sympathy and assistance in the work which he has attempted. He has visited the offices of a great many dentists, but he never yet examined one where there was not the evidence of some bit of special knowledge, some wisdom, the fruit of a particular experience, which the profession at large would be the better for, and they themselves, were it written out, none the poorer. If dentistry is to remain a profession, or a branch of a profession, it must have its literature, which can only be made perfect through each member furnishing material to the extent of his ability. Communications are therefore invited from every earnest man who really has anything to say. W. C. BARRETT.

BUFFALO, N. Y., December 15, 1882.

